# Nanomaterials and Microorganisms: From Green Synthesis to Antibacterial Applications in Medicine and Agriculture

**DOI:** 10.3390/nano12234265

**Published:** 2022-11-30

**Authors:** Monika Mortimer, Anne Kahru

**Affiliations:** 1 Institute of Environmental and Health Sciences, College of Quality and Safety Engineering, China Jiliang University, Hangzhou 310018, China; 2 National Institute of Chemical Physics and Biophysics (NICPB), Laboratory of Environmental Toxicology, Akadeemia tee 23, 12618 Tallinn, Estonia; 3 Estonian Academy of Sciences, Kohtu 6, 10130 Tallinn, Estonia

Nanomaterial-based solutions for microorganism-related issues are gaining interest in medical fields, consumer applications, and agriculture. The main issues driving innovation in this field are the increasing antimicrobial resistance (AMR) of conventional antibiotics and the environmental toxicity of widely used agricultural chemicals—a problem that has been largely overlooked. Therefore, the synthesis of new materials or modification of the existing ones is urgently needed to tackle these health and environmental problems. The top priority of these issues was reflected by the topics of the research papers submitted to the Special Issue “Nanomaterials and Microorganisms”. Namely, the common denominator of the papers collected in this Special Issue was the design of innovative nanomaterials that showed improved activity against pathogenic bacteria. The application fields of these materials ranged from medicine to agriculture and the proposed novel materials varied from nanocomposites based on traditional antibacterial nanomaterials such as silver or zinc oxide to selenium-based or reduced graphene oxide/S/Se nanocomposites.

One of the increasingly used approaches to produce nanomaterials with efficient antibacterial properties is using a green synthesis process that involves either plant extracts or living microorganisms. During this synthesis process, which is applicable to metal-based nanomaterials, the resulting materials are rendered highly antibacterial either by a coating or structural incorporation of bioactive compounds originating from the biological materials used for synthesis. Such an approach was used by Ahmed et al. to biologically synthesize ZnO nanoparticles (NPs) in the culture supernatants of *Bacillus cereus*, a bacterial strain isolated from soils of rice fields at Zhejiang University, Hangzhou, China [1]. The biosynthesized ZnO NPs effectively eradicated bacterial pathogens known to cause bacterial panicle blight in rice. Thus, it was demonstrated that green synthesis is a promising method for the production of novel nanopesticides. Sitohy et al. developed a different green synthesis approach by combining Ag NPs, which are well known for their antibacterial efficiency, with plant seed proteins to produce nanocomposites with higher antibacterial efficiency than each of the single components [2]. The proteins acted as stabilizers of Ag and also increased the antibacterial potency of the nanocomposite, plausibly due to positively charged and hydrophobic amino acid residues which facilitated contact with the bacterial cell.

Charge-dependent antibacterial activity of NPs is a key issue that needs to be considered in the design of new nanomaterials. This topic was addressed by Vihodceva et al. who demonstrated that positively charged spherical hematite (α-Fe_2_O_3_) NPs specifically interacted with the membrane of Gram-negative *Escherichia coli* and exerted antibacterial effects [3]. Negatively charged α-Fe_2_O_3_ NPs did not have antibacterial properties. Interestingly, the positive α-Fe_2_O_3_ NPs selectively acted on Gram-negative bacteria, but not on Gram-positive *Staphylococcus aureus*. Both the above-mentioned bacteria are clinically relevant models for the evaluation of novel antibacterial compounds. Furthermore, the NPs did not affect the bioluminescence of the naturally luminescent marine bacteria *Vibrio fischeri* (EC_50_ > 1000 mg/L) which suggests that these NPs would be safe in environmental applications.

Other ways to develop environmentally compatible effective antibacterial NPs include the use of natural polysaccharides for the synthesis of nanocomposites. Perfileva et al. synthesized selenium (Se) nanocomposites based on natural polysaccharide matrices arabinogalactan, starch, and kappa-carrageenan which were shown to increase potato productivity [4]. Such stimulating effect on plant growth was connected to the nanocomposite impacts on the rhizosphere bacteria, where suppression of pathogenic or stimulation of beneficial bacteria could influence plant health. The authors concluded that the positive effect of natural matrix-based Se nanocomposites on plants contributes to the future application of these nanomaterials on rhizosphere bacteria and to increasing the stress resistance of crop plants.

In environmental applications as well as consumer products, including medical settings, antibacterial surfaces are considered beneficial for eradicating pathogenic bacteria. Nanotechnology is considered an innovative approach to improving the efficiency of such surfaces. Specifically, nano-enabled photocatalytically active coatings are being developed and improved to render surfaces antibacterial. As reported by Vihodceva et al., a combination of silver and hematite NPs as a Ag/AgCl/α-Fe_2_O_3_ composite embedded into ethylene-co-vinyl acetate coating yielded an effective photocatalytic material which distinctly and rapidly inhibited bacterial growth whereas reactive oxygen species, and not the shedding of Ag-ions, played the main role in the photocatalytic bacterial inactivation [5]. In addition to having high photocatalytic activity under visible light, the nanocomposite coating was highly durable through several photocatalytic and washing cycles.

The photocatalytic mode of action of antimicrobial nanomaterials is not always applicable in human medicine and thus different approaches must be used. For example, Nobre et al. demonstrated that hydroxyapatite which is a calcium–phosphate ceramic material with known anti-adhesive properties, functioned well as an antibiofilm material when used as a nanosuspension [6]. The study showed that a pure hydroxyapatite-based mouthwash was a promising method for oral biofilm management with a low risk for side effects and AMR development commonly observed in the case of traditional antibacterial agents.

AMR-related issues were addressed in two papers of the Special Issue. Brar et al. developed nano-encapsulated drugs consisting of chitosan and silica NPs containing tetracycline and chlorpromazine [7]. The encapsulation of the antibiotic drug and efflux pump inhibiting agent into NPs improved their antibacterial efficacy due to targeting AMR mechanisms of pathogenic bacteria and reducing intracellular pathogen load. The study demonstrated that NP-enabled combination therapy has promising prospects in combating multidrug-resistant pathogens. Niranjan et al. used a carbon-based antibacterial nanomaterial, namely, reduced graphene oxide (rGO), and deposited sulfur or a combination of sulfur and selenium on it to create antibacterial NPs with superior characteristics [8]. Among the three synthesized NPs, rGO-S/Se exhibited especially strong antibacterial effects against Gram-positive pathogens *S. aureus* and *Enterococcus faecalis* (growth inhibition >90% at 200 mg/L) due to membrane damage and oxidative stress.

In summary, the papers in this collection have proposed several new innovative nanomaterials with different modes of action against bacteria, thereby contributing to finding new solutions for combating the health issues of current antimicrobials. In addition to developing efficient antibacterial NPs, the impacts on the beneficial bacteria and microbiota need to be considered. The research in this direction is gaining momentum, as also illustrated by the recent publications in *Nanomaterials* [9]. Combining material development with safety assessment is the key to designing safer nanomaterials and ensuring the sustainability of novel innovative nanomaterial-based applications related to microorganisms.
